# Association of recommended food score with depression, anxiety, and quality of life in Korean adults: the 2014–2015 National Fitness Award Project

**DOI:** 10.1186/s12889-019-7298-8

**Published:** 2019-07-17

**Authors:** Jo-Eun Lee, You Jin Kim, Hee Jung Park, Saejong Park, Hyesook Kim, Oran Kwon

**Affiliations:** 1 Department of Clinical Nutrition Science, The Graduate School of Clinical Health Sciences, Ewha Womans University, Seoul, Republic of Korea; 2 Department of Nutritional Science and Food Management, Ewha Womans University, 52, Ewhayeodae-gil, Seodaemun-gu, Seoul, 03760 Republic of Korea; 3 Department of Food and Nutrition, Yuhan University, Bucheon, Republic of Korea; 4 Department of Sport Science, Korea Institute of Sport Science, Seoul, Republic of Korea

**Keywords:** Recommended food score: diet quality: depression: anxiety: quality of life

## Abstract

**Background:**

A healthy diet is a key determinant of an individual’s health status and is closely related to mental health and quality of life (QoL); however, the exact nature of the relationship is unknown. This study hypothesized that a higher diet quality score is associated with a lower observance of symptoms of depression and anxiety and a higher QoL.

**Methods:**

This study evaluated 1,295 adults (521 men; 774 women) aged 19–64 years, who participated in the 2014–2015 National Fitness Award Project. Diet quality was measured by the recommended food score (RFS), and mental health and QoL were assessed by the beck depression inventory (BDI), beck anxiety inventory (BAI), and the World Health Organization QoL–Brief (WHOQoL–BREF).

**Results:**

After adjusting for covariates, the individuals with depression had a significantly lower RFS value compared to those without depression, and the group with a QoL score above the median had a higher RFS value than the group with a QoL score below the median. These trends occurred in both men and women. Subjects in the highest tertile of RFS showed a lower odds of depression (OR = 0.51, 95% CI = 0.32–0.81, *p*-trend = 0.0043) and a QoL score below the median (OR = 0.40, 95% CI = 0.30–0.54, *p*-trend < 0.0001) compared with those in the lowest tertile. The RFS was not associated with anxiety.

**Conclusions:**

Our data suggest that higher diet quality may be associated with lower depressive symptoms and a better QoL in Korean adults.

## Background

Advances in science and technology over recent years have led to an increased life expectancy and improved living standards. As a result of this gain in life expectancy, there has been increased attention toward physical, mental, and health status, accompanied by a marked interest in the quality of life (QoL). While there is no universal consensus on the definition of QoL, the World Health Organization (WHO) defines QoL as “an individual’s perception of their position in life in the context of the culture and the value system where they live, and in relation to their goals, expectations, standards and concerns” [1]. In its broadest concept, QoL encompasses physical, mental, social, and disease domains [2], and these domains are related to an increased risk of mortality [3]. The mental domain includes depression and anxiety [2], and depression and anxiety negatively influence QoL [4–6].

QoL is a relative concept that can be changed according to the degree of development of politics, the economy, society, and individual characteristics of social members, living conditions, values, and customs [7]. The pattern and extent of depression also vary considerably among individuals, owing to influences of genetic factors, gender, the social environment, and lifestyle differences [8]. Proper eating habits are key determinants of an individual’s physical and mental health status and are closely related to QoL [9]. In the same regard, irregular eating habits not only hamper physical health but also affect an individual’s psychological state and emotional stability [10]. Research on the association between diet and depression indicates that intakes of isolated nutrients or foods, such as omega-3 unsaturated fatty acids, folic acid, and zinc, iron or copper are involved in decreasing the risk of depression [11–15].

The limitation of focusing on the association of individual nutrients or particular food groups with diseases is the lack of consideration for the complicated interactions and cumulative effects that seem to be responsible for disease epidemics [16]. Since diet is a multidimensional exposure, it is difficult to conclude which nutrient or food is responsible for the prevalence rate of a particular disease or symptomatology [17]. Hence, in the last few years, studies have shifted toward measuring overall dietary patterns. An accumulating number of studies have shown that associations exist between overall dietary quality and depression, anxiety, or QoL, in adults in western countries [18–28]. Findings from cross-sectional investigations suggested connections between the healthy dietary pattern score [18], healthy eating index (HEI) score [19–21], and dietary diversity score [22] with depression or anxiety, and the Mediterranean diet score (MDS) with self-perceived health-related QoL (HRQoL) [23]. Cohort studies have demonstrated the influence of the MDS on mental and physical HRQoL [24], the total diet score on HRQoL [25], and the recommended food score (RFS) on HRQoL [26]. Recently, several randomized controlled trials (RCT) [27, 28] conducted in Australia indicated that a Mediterranean-style diet could improve mental health in adults with depression. However, the results of these investigations conducted in western populations may not apply to Korean people due to the differences in dietary habits.

Our research team previously examined the association between dietary quality and oxidative stress in the Korean population [29]. The RFS used in that study had been modified to measure the diet quality of Koreans consuming high amounts of antioxidant nutrients [29]. This same tool was used to clarify the correlation of depression with health and nutritional intake in Korean adult women aged 50–64 years, using data from the 2013 Korea National Health and Nutrition Examination Survey (KNHANES) [30]. However, the information used in that research was obtained from an unstructured questionnaire (“yes” or “no” responses to questions, such as “Do you currently have depression?”) and only analyzed the unadjusted data [30]. Despite the possibility that RFS, a diet quality index that appears to be associated with lowering oxidative stress, may be related to mental health or QoL and that the expected results would aid health and nutrition promotion among people, to the best of our knowledge, no studies have investigated this association overall. Therefore, the present research reassessed the association of the RFS with depression, anxiety, and QoL in Korean adult men and women, using data sourced from structured information.

## Methods

### Study subjects

This study was performed within the framework of a cross-sectional survey designed to develop criteria referencing health-related fitness standards for the National Fitness Award Project [31]. The National Fitness Award project is a large-scale national project currently managed by 21 centers. The Korea Institute of Sports Science has been conducting this project since 2012, with the support of the Ministry of Culture, Sports, and Tourism to promote health by exercise, physical, and sporting activities in daily life. Adults and seniors have been recruited since 2014, and the present study was based on the 2014–2015 National Fitness Award Project for participants aged 19–64 years (*n* = 2,280). We excluded pregnant subjects (*n* = 1); those without data for the RFS (*n* = 973); those without data on the beck depression inventory (BDI), beck anxiety inventory (BAI), World Health Organization Quality of Life–Brief (WHOQoL–BREF), or all three indicators (*n* = 8); and those lacking background information, such as age, gender, and body mass index (BMI) (*n* = 3). Finally, 1,295 subjects were eligible for further analysis and stratified by gender. Ethical approval of this retrospective study was given by the Institutional Review Board (IRB) of the Korea Institute of Sport Science (KISS-201504_EFS-002-01), and Ewha Womans University (120–2, August 2018), and all subjects provided their written informed consent to participate in the study.

### General characteristics, socioeconomic characteristics, and anthropometric measurements

Participants were interviewed by trained interviewers to obtain general information on demographic and socioeconomic characteristics, medical history, and health-related behaviors, including age, family income, education, marital status, smoking behavior, alcohol consumption, and menopausal state. The marital status was classified as with and without a spouse and included divorced, separated, disconnected, and single. Participants who have smoked or quit smoking within the past 12 months were considered to be smokers. Participants who consumed alcohol more than once per month were regarded as drinkers. Those who were diagnosed with one or more chronic diseases were considered to have chronic diseases. Physical activity was assessed through a Korean version of the International Physical Activity Questionnaire (IPAQ) short form [32]. The participants were asked to record how many hours they participated in various physical activities, and the level of physical activity was quantified as metabolic equivalent task-hours per week (MET-h/week) [33]. Standing height and body weight were measured using a stadiometer (Seca Corporation, Columbia, MD, USA) and an electronic weight scale (Inbody 720, Biospace, Seoul, Korea), respectively. BMI was computed as body weight divided by the square of the body height (kg/m^2^). Waist circumference was measured midway between the lowest rib and the iliac crest, using a tape measure (Tanita anthropometric tape, Seoul, Korea).

### Dietary assessment

Diet quality score was assessed using the RFS developed by Kant et al. [34] to measure overall diet quality, and modified and validated by Kim et al. [29] for the reported consumption of foods based on the Korean dietary guidelines. The RFS was assessed using a questionnaire related to the previous 12 months. Forty-six foods or food groups corresponding to recommended food groups were included: grains (1), legumes (4), vegetables (17), seaweeds (2), fruits (12), fish (5), dairy products (3), nuts (1), and tea (1). The questions had structured answer choices of yes/no to whether each food item was consumed at least once a week. The daily frequency of meals was also recorded. Participants were allocated a score of 1 if they consumed the recommended food at least weekly or ate three meals daily on a regular basis. Thus, the maximum possible score is 47, and a higher score indicates a better diet quality.

### Depression assessment

The BDI is a 21-item self-report instrument that was developed to measure symptoms of depression [1, 35]. The current study implemented a standardized Korean version of the BDI developed by Hahn et al. [36], which contains items related to the affective, cognitive, motivational, and physiological symptoms of depression. Each item is rated by the individual on a 4-point Likert-type scale, with 0 meaning “not at all” and 3 indicating “severely,” for how much the symptoms were experienced in the past week. Thus, the maximum possible score is 63, and a high score indicates a severe depression status.

### Anxiety assessment

The BAI, originally released in 1988 [37], has been regarded as one of the most popular screening and outcome research tools for measuring self-reported anxiety symptomatology [38–41]. In this study, a standardized Korean version of the BAI, developed by Kwon et al. [42], was used. It measures 21 items on a 4-point Likert-type scale for the occurrence of sensations related to anxiety (e.g., not at all, a little, moderate, and severe), focusing both on cognitive and somatic symptoms. Thus, the maximum possible score is 63, and a higher score indicates a severe anxiety status.

### QoL assessment

QoL was assessed using the validated Korean version of the WHOQoL–BREF [43], which is a short (26-item) version of the original WHOQoL–100 questionnaire [44]. It uses a 5-point Likert scale to generate a profile and score for each of four domains: physical health (7 items), psychological health (6), social relationships (3), and environment (8). The higher scores indicate better satisfaction with life and well-being, except in regards to three negative facets, which include pain and discomfort, need for medical treatment, and negative feelings.

### Statistical analysis

Participant’s characteristics were shown as mean and standard deviation for continuous variables, and as numbers and percentages for categorical variables. Significant differences were evaluated between men and women, and tertiles of the RFS, respectively, using the chi-square test for categorical variables, and the Student’s *t*-test for continuous variables. Pearson’s correlation test was used to analyze correlations between RFS and depression, anxiety, and QoL. Multiple linear regression analysis was used to investigate the associations between the RFS and depression, anxiety, and QoL after adjusting for covariates. Subjects were dichotomized into two groups according to the presence of depression (BDI, yes: score ≥ 16; no: score < 16 [42, 45]) and anxiety (BAI, yes: score ≥ 22; no: score < 22 [37, 46]), and the WHOQOL-BREF was divided into two groups using the median (QoL score below the median: < 82 points; QoL score above the median: ≥82 points [47]) since there was no specific criterion. A general linear model was used to analyze the differences in RFS values among the two groups after adjusting for covariates. Multiple logistic regression analysis was used to estimate the adjusted odds ratios (ORs), and its corresponding 95% confidence intervals (CIs) indicated whether the RFS was linked with depression, anxiety, and a QoL score below the median (the lowest RFS was the reference category). The covariates in this study were statistically significant in univariate analyses or were known to be factors associated with mental health or QoL in the existing literature. All analyses were adjusted for age and gender (model 1) and, additionally, for BMI, marital status, smoking, drinking, physical activity, and diagnosis of one or more chronic diseases (model 2). All statistical analyses were performed using IBM SPSS Statistics v.21 software. Statistical significance was set at *p* < 0.05.

## Results

### Characteristics of the participants

Table 1 displays the characteristics of the studied subjects. The participants had a mean age of 44.0 ± 14.5 years, and 59.7% were women. Overall, the women had a lower BMI (23.6 ± 3.2 kg/m^2^), had attained a lower educational qualification (less than middle school 21.9%), were married (74.7%), had chronic diseases (24.0%), drank alcohol less than once per month (50.6%) and were non-smokers (99.2%) when compared with the male subjects. The women also had a higher RFS (*p =* 0.0007), poor QoL score (*p =* 0.0001), and were less depressed (BDI; *p* < 0.0001) and less anxious (BAI; *p* < 0.0001) compared with the men.

**Table 1:** General characteristics of the subjects according to gender

	Total	Men	Women	*p*-value
	(*n* = 1,295)	(*n* = 521)	(*n* = 774)	
Age (year)	44.0 ± 14.5	38.5 ± 15.1	47.7 ± 12.8	< 0.0001
Age group				
< 20	42 (3.2%)	38 (7.3%)	4 (0.5%)	< 0.0001
20–29	248 (19.2%)	155 (29.8%)	93 (12.0%)	
30–39	208 (16.1%)	88 (16.9%)	120 (15.5%)	
40–49	225 (17.4%))	82 (15.7%)	143 (18.5%)	
≥ 50	572 (44.2%)	158 (30.3%)	414 (53.5%)	
Height (cm)	163.9 ± 9.2	172.3 ± 6.6	158.2 ± 5.7	< 0.0001
Weight (kg)	65.4 ± 12.5	74.7 ± 11.7	59.1 ± 8.4	< 0.0001
BMI (kg/m^2^)	24.2 ± 3.3	25.1 ± 3.3	23.6 ± 3.2	< 0.0001
Waist circumference (cm)	84 ± 9.0	86.8 ± 8.9	82.1 ± 8.6	< 0.0001
Education				
≤ Middle school	130 (15.0%)	27 (6.8%)	103 (21.9%)	< 0.0001
High school	216 (24.8%)	76 (19.0%)	140 (29.8%)	
≥ College	524 (60.2%)	297 (74.3%)	227 (48.3%)	
Income per month (10,000won)				
≤ 200	356 (27.6%)	111 (21.4%)	245 (31.7%)	0.0002
201–400	547 (42.3%)	245 (47.2%)	302 (39.1%)	
> 400	389 (30.1%)	163 (31.4%)	226 (29.2%)	
Marital status				
With spouse	852 (65.8%)	274 (52.6%)	578 (74.7%)	< 0.0001
Without spouse	443 (34.2%)	247 (47.4%)	196 (25.3%)	
Smoking				
Yes	140 (10.8%)	134 (25.7%)	6 (0.8%)	< 0.0001
No	1155 (89.2%)	387 (74.3%)	768 (99.2%)	
Alcohol drinking				
< 1 time/month	462 (38.4%)	110 (21.7%)	352 (50.6%)	< 0.0001
≥ 1 time/month	742 (61.6%)	398 (78.3%)	344 (49.4%)	
Physical activity (MET-hr/wk)	16.3 ± 16.2	16.5 ± 16.4	16.2 ± 16.2	0.7378
Diagnosis of one or more chronic disease				
Yes	294 (22.7%)	108 (20.7%)	186 (24.0%)	0.1490
No	1001 (77.3%)	413 (79.3%)	588 (76.0%)	
Menopausal status				
Yes	209 (46.2%)	–	209 (46.2%)	
No	243 (53.8%)	–	243 (53.8%)	
RFS	25.3 ± 9.3	24.3 ± 9.7	26.1 ± 9.0	0.0007
BDI	8.3 ± 5.9	7.3 ± 5.6	9.0 ± 6.1	< 0.0001
BAI	6.1 ± 6.6	5.2 ± 6.1	6.8 ± 6.9	< 0.0001
WHOQOL	83.0 ± 12.2	84.6 ± 12.8	81.9 ± 11.6	0.0001

Abbreviations: *BMI* body mass index, *MET* metabolic equivalent task, *RFS* recommended food score, *BDI* beck depression inventory, *BAI* beck anxiety inventory; *WHOQOL* The World Health Organization quality of life assessment

Data were available for 870 subjects for education; 1,292 subjects for income; 1,204 subjects for alcohol drinking; 452 subjects for menopausal status; 1,265 subjects for BDI; 1,262 subjects for BAI; and 1,257 subjects for WHOQOL

Values are expressed as N (%) or mean ± SD

Student’s t-test or Chi-square tests for continuous and categorical variables, respectively, between men and women

### Association of the RFS with depression, anxiety, and QoL

Table 2 shows the correlation of the diet quality score (RFS) with depression (BDI), anxiety (BAI), and QoL (WHOQoL-BREF). The RFS was negatively correlated with the BDI scores and positively correlated with the WHOQoL-BREF scores. Table 3 shows the association of the diet quality score (RFS) with depression (BDI), anxiety (BAI), and QoL (WHOQoL-BREF). Overall, after adjustment for all potential confounding factors, a better diet quality (RFS) was associated with lower depression (β = − 0.16, *p* < 0.0001) and better QoL (β = 0.21, *p* < 0.0001). The RFS had a significant positive association with physical health, psychological health, social relationship, and environment (*p* < 0.0001 for all) but it was not significantly associated with anxiety (*p* = 0.0898). The multiple linear regression analysis conducted separately by gender, showed that a healthier diet quality score (higher RFS) was associated with a lower depression score (men: *p* = 0.0026, women: *p* < 0.0001) and better QoL (men: *p* = 0.0001, women: *p* < 0.0001) in both men and women after adjustment for all potential confounding factors. However, in men, there was no significant association between a healthier diet quality score (higher RFS) and social relationships (*p* = 0.2678).

**Table 2:** Correlation between RFS and depression, anxiety and quality of life

	Total		Men		Women	
	R	p-value	R	p-value	R	p-value
BDI	−0.16	< 0.0001	−0.16	0.0003	−0.20	< 0.0001
BAI	−0.05	0.7677	−0.05	0.2711	− 0.07	0.0556
WHOQOL-BREF	0.21	< 0.0001	0.20	< 0.0001	0.25	< 0.0001
WHO Domain						
Physical health	0.14	< 0.0001	0.14	0.0012	0.16	< 0.0001
Psychological	0.20	< 0.0001	0.16	< 0.0001	0.23	< 0.0001
Social relationship	0.13	< 0.0001	0.06	0.1736	0.20	< 0.0001
Environment	0.20	< 0.0001	0.18	< 0.0001	0.23	< 0.0001
Total_WHODOM	0.19	< 0.0001	0.17	0.0002	0.24	< 0.0001

Abbreviations: *RFS* recommended food score, *BDI* beck depression inventory, *BAI* beck anxiety inventory; *WHOQOL* WHO quality of life, *DOM* domain

Data were available for 1265 subjects (for BDI); 1262 subjects (for BAI); 1257 subjects (for WHOQOL)

**Table 3:** Association of RFS with depression, anxiety and quality of life

	Total			Men			Women		
	β	SE	p-value	β	SE	p-value	β	SE	p-value
BDI									
Model 1	−0.19	0.01	< 0.0001	−0.17	0.02	0.0001	−0.21	0.02	< 0.0001
Model 2	−0.16	0.02	< 0.0001	−0.14	0.03	0.0026	−0.18	0.03	< 0.0001
BAI									
Model 1	−0.07	0.02	0.0104	−0.07	0.02	0.1160	−0.07	0.02	0.0449
Model 2	−0.05	0.02	0.0898	−0.04	0.03	0.3808	−0.06	0.03	0.1633
WHOQOL-BREF									
Model 1	0.25	0.03	< 0.0001	0.22	0.05	< 0.0001	0.28	0.04	< 0.0001
Model 2	0.21	0.04	< 0.0001	0.18	0.06	0.0001	0.25	0.05	< 0.0001
WHO Domain									
Physical health									
Model 1	0.19	0.00	< 0.0001	0.17	0.01	< 0.0001	0.21	0.01	< 0.0001
Model 2	0.15	0.01	< 0.0001	0.14	0.01	0.0027	0.17	0.01	< 0.0001
Psychological									
Model 1	0.24	0.00	< 0.0001	0.22	0.01	< 0.0001	0.25	0.01	< 0.0001
Model 2	0.20	0.01	< 0.0001	0.19	0.01	< 0.0001	0.21	0.01	< 0.0001
Social relationship									
Model 1	0.17	0.00	< 0.0001	0.08	0.01	0.0475	0.23	0.00	< 0.0001
Model 2	0.13	0.01	<0.0001	0.05	0.01	0.2678	0.20	0.01	< 0.0001
Environment									
Model 1	0.25	0.00	< 0.0001	0.21	0.01	< 0.0001	0.27	0.00	< 0.0001
Model 2	0.21	0.01	< 0.0001	0.18	0.01	0.0001	0.23	0.01	< 0.0001
Total WHODOM									
Model 1	0.24	0.02	< 0.0001	0.20	0.04	< 0.0001	0.28	0.03	< 0.0001
Model 2	0.20	0.03	< 0.0001	0.16	0.04	0.0005	0.24	0.03	< 0.0001

*RFS* Recommended food score, *BDI* beck depression inventory, *BAI* beck anxiety inventory, *WHOQOL* The World Health Organization quality of life assessment, *DOM* domain

Data were available on 1,265 subjects for BDI; 1,262 subjects for BAI; and 1,257 subjects on WHOQOL

Model 1 adjusted for age

Model 2 additionally adjusted BMI, marital state, smoking, drinking, physical activity, and diagnosis of one or more chronic disease

### RFS values of the subjects by depression, anxiety, and QoL

Table 4 gives the adjusted mean scores for the RFS, according to the presence of depression, anxiety, and a QoL score below the median. After controlling for all covariates, participants who reported no depressive symptoms had a significantly higher diet quality score among the total subjects (*p* = 0.0129). These results show that marginally significant trends exist in the results of men (*p =* 0.0769) and women (*p =* 0.0788), respectively. However, there were no significant differences between the non-anxiety group and anxiety group (*p* = 0.9376). The group with a QoL score above the median had a higher diet quality score than the group with a QoL score below the median after controlling for all covariates, regardless of gender (*p* < 0.0001 for all).

**Table 4:** RFS values of the subjects by depression, anxiety and quality of life

	Total				Men				Women			
	*n*	RFS values	*p*-value		*n*	RFS values	*p*-value		*n*	RFS values	*p*-value	
			Model 1	Model 2			Model 1	Model 2			Model 1	Model 2
BDI												
Non-Depression	1109	25.61 ± 9.32	0.0002	0.0129	468	24.54 ± 9.78	0.0140	0.0769	641	26.40 ± 8.89	0.0040	0.0788
Depression	156	23.33 ± 9.56			45	21.38 ± 8.99			111	24.13 ± 9.71		
BAI												
Non-Anxiety	1218	25.31 ± 9.46	0.7867	0.9376	501	24.25 ± 9.69	0.8838	0.6117	717	26.04 ± 9.23	0.6735	0.6700
Anxiety	44	25.55 ± 7.21			12	25.33 ± 11.45			32	25.63 ± 5.07		
WHOQOL												
Above the median	667	27.04 ± 9.40	< 0.0001	< 0.0001	295	26.18 ± 9.72	< 0.0001	< 0.0001	372	27.73 ± 9.08	< 0.0001	< 0.0001
Below the median	590	23.54 ± 8.97			211	21.82 ± 9.11			379	24.50 ± 8.76		

*RFS* recommended food score, *BDI* beck depression inventory, *BAI* beck anxiety inventory, *WHOQOL* The World Health Organization quality of life assessment

Subjects were dichotomized into 2 groups according to the presence of depression (BDI ≥16 scores [42, 45]) and anxiety (BAI ≥22 scores [37, 46]) and the median value of WHOQOL-BREF (QoL < 82 points [47])

Model 1 adjusted for age

Model 2 additionally adjusted BMI, marital state, smoking, drinking, physical activity, and diagnosis of one or more chronic disease

### OR of depression, anxiety, and QoL according to the RFS tertiles

The associations of the RFS with depression, anxiety, and QoL, estimated by multiple logistic regression models, are presented in Table 5. Among the total participants, diet quality score (RFS) was significantly associated with a lower risk of depression (OR per 1 score increase: 0.97; 95% CI: 0.98–0.99) and a QoL score below the median (OR per 1 score increase: 0.96; 95% CI: 0.94–0.97) in the fully adjusted models, respectively. There was no association between diet quality score and odds of anxiety.

**Table 5:** Odds ratio and 95% CI of depression, anxiety and quality of life score below the median according to the tertiles of RFS

	Total						Men							Women				
	*n*	Model 1		*n*	Model 2		*n*	Model 1		*n*	Model 2		*n*	Model 1		*n*	Model 2	
Depression																		
Continuous	1265	0.96	(0.94–0.98)	1176	0.97	(0.95–0.99)	513	0.96	(0.92–0.99)	500	0.97	(0.94–1.00)	752	0.97	(0.94–0.98)	676	0.98	(0.95–1.00)
Tertile 1	471	1.00 (Reference)		440	1.00 (Reference)		216	1.00 (Reference)		211	1.00 (Reference)		255	1.00 (Reference)		229	1.00 (Reference)	
Tertile 2	374	0.63	(0.42–0.95)	350	0.71	(0.46–1.09)	142	0.50	(0.23–1.08)	137	0.64	(0.29–1.40)	232	0.70	(0.43–1.15)	213	0.77	(0.46–1.31)
Tertile 3	420	0.44	(0.29–0.68)	386	0.51	(0.32–0.81)	155	0.31	(0.13–0.73)	152	0.36	(0.14–0.89)	265	0.51	(0.31–0.85)	234	0.59	(0.34–1.03)
p-trend		0.0002			0.0043			0.0042			0.0232			0.0093			0.0635	
Anxiety																		
Continuous	1262	1.00	(0.96–1.02)	1174	1.00	(0.96–1.03)	513	1.00	(0.94–1.06)	500	1.02	(0.95–1.09)	749	0.99	(0.95–1.03)	674	0.99	(0.95–1.03)
Tertile 1	469	1.00 (Reference)		439	1.00 (Reference)		215	1.00 (Reference)		210	1.00 (Reference)		254	1.00 (Reference)		229	1.00 (Reference)	
Tertile 2	372	1.41	(0.68–2.92)	348	1.46	(0.69–3.08)	141	0.24	(0.03–2.00)	136	0.32	(0.04–2.75)	231	2.21	(0.92–5.32)	212	2.17	(0.89–5.30)
Tertile 3	421	0.90	(0.41–1.98)	387	0.89	(0.39–2.08)	157	0.97	(0.27–3.40)	154	1.27	(0.31–5.27)	264	0.92	(0.33–2.53)	233	0.85	(0.29–2.47)
p-trend		0.7939			0.8321			0.8890			0.8477			0.8229			0.7668	
QOLscore below the median																		
Continuous	1257	0.95	(0.94–0.96)	1171	0.96	(0.94–0.97)	506	0.95	(0.93–0.97)	494	0.95	(0.93–0.97)	751	0.95	(0.93–0.97)	677	0.96	(0.94–0.98)
Tertile 1	464	1.00 (Reference)		435	1.00 (Reference)		210	1.00 (Reference)		206	1.00 (Reference)		254	1.00 (Reference)		229	1.00 (Reference)	
Tertile 2	373	0.63	(0.47–0.83)	350	0.67	(0.50–0.90)	143	0.59	(0.38–0.91)	138	0.66	(0.42–1.04)	230	0.65	(0.45–0.94)	212	0.68	(0.46–1.01)
Tertile 3	420	0.35	(0.26–0.47)	386	0.40	(0.30–0.54)	153	0.33	(0.21–0.53)	150	0.37	(0.23–0.60)	267	0.37	(0.25–0.53)	236	0.42	(0.28–0.63)
p-trend		< 0.0001			< 0.0001			< 0.0001			< 0.0001			< 0.0001			< 0.0001	

Abbreviations: *RFS* recommended food score, *QOL* WHO quality of life

Model 1: adjusted for age

Model 2: additionally adjusted BMI, smoking, drinking, chronic disease, marital state, physical activity

Depression: cut-off point is 16 point [42, 45], Anxiety: cut-off point is 22 point [37, 46], QOL: cut-off point is 82 point (median point) [47]

Continuous: per RFS 1 point increment

Data were available for 1265 subjects (for BDI); 1262 subjects (for BAI); 1257 subjects (for WHOQOL)

In the age-adjusted models, compared to the group with the highest tertile of RFS, the OR for depression in the group with the lowest tertile was 0.31 (95% CI: 0.13–0.73, *p*-trend 0.0042) and 0.51 (95% CI: 0.31–0.85, *p*-trend 0.0093) for men (*n* = 513) and women (*n* = 752), respectively. Compared to the group with the highest tertile of RFS, the OR of having a QoL score below the median for the group with the lowest tertile was 0.33 (95% CI: 0.21–0.53, *p*-trend < 0.0001) and 0.37 (95% CI: 0.25–0.53, *p*-trend < 0.0001) for men (*n* = 506) and women (*n* = 751), respectively.

In the fully adjusted models, compared to the group with the highest tertile of RFS, the OR for depression in the group with the lowest tertile was 0.36 (95% CI: 0.14–0.89, *p*-trend 0.0232) and 0.59 (95% CI: 0.34–1.03, *p*-trend 0.0635) for men (*n* = 500) and women (*n* = 679), respectively. Compared to the group with the highest tertile of RFS, the OR of having a QoL score below the median for the group with the lowest tertile was 0.37 (95% CI: 0.23–0.60, *p*-trend < 0.0001) and 0.42 (95% CI: 0.28–0.63, *p*-trend < 0.0001) for men (*n* = 494) and women (*n* = 677), respectively.

## Discussion

This study found that a higher RFS was associated with less severe depressive symptoms and a better QoL in Korean adults. These associations of RFS with depression and QoL were observed in men and women, respectively. It is the first comprehensive study to identify a positive relationship between the RFS and mental health and QoL in Korean adults [48].

There is only one previous article [30] that examined the influence of diet quality of Koreans on depression using a modified RFS, which is the same dietary quality index used in the current study. While it was found that Korean adult women aged 50–64 years with depression had a lower RFS than those with no depressive symptoms, the study had some limitations (an unstructured questionnaire with a mere yes or no answer to depression, and a relevance analysis that did not consider covariates at all) [30]. Applying a different RFS tool, the Australian Longitudinal Study on Women’s Health (ALSWH) [49] conducted in middle-aged women investigated the relationship between an RFS for Australians and depression. After a 9-year follow-up, long-term maintenance of good diet quality was suggested to be associated with a reduced odds of depression. However, based on their 12-year follow-up study, these same researchers [50] revealed that initial associations emerged alongside baseline measures of diet quality, while depressive symptoms disappeared when methods handling time-varying covariates were applied. This suggests that residual confounding might have impacted previous studies that had established a connection between diet and depression.

As far as we know, to date, the interaction between the RFS and QoL has only been reported in the Wellbeing, Eating and Exercise for a Long Life (WELL) study conducted in Australian adults aged 55–65 years, in which adults with better quality diets reported better HRQoL, and additional associations with emotional wellbeing in women [26]. Our study and existing literature suggest a healthier diet quality is associated with improved mental health and QoL among Korean adults. However, these observational studies may have confounders, whereby subjects in better health or with a better QoL may have self-endorsed or selected to perform a physical exercise or consume nutrient-rich foods at the time of measurement. Therefore, further prospective investigations or intervention research is needed to confirm whether the association truly represents a cause–effect relationship. A recent RCT reported that application of structured dietary support, focused on elevating diet quality by using a modified Mediterranean diet model, would be more effective in reducing the severity of depressive symptomatology than would creating a social support control condition (befriending) [27]. Another recent RCT reported that a Mediterranean-style diet supplemented with fish oil could improve mental health in adults suffering depression [28]. In the future, more systematic RCT studies on the relationship between RFS and mental health and QoL should be conducted.

Diet quality indices can not only evaluate compliance with recommended dietary guidelines but also certain diet types, such as the Mediterranean diet [17]. Previous studies have concluded that the association between scores in diet quality (RFS and alternative-MDS) and nutrient intake are possibly explained by the guidelines and features of the Mediterranean diet, which is significantly comprised of plant foods containing potentially high amounts of active dietary antioxidants [29]. High adherence to the Mediterranean diet was considered important in improving the HRQoL, according to the Seguimiento Universidad de Navarra (SUN) cohort study [24]. Cross-sectional surveys of the Spanish adult population showed that better self-perceived mental and physical QoL were related to greater compliance with the Mediterranean diet [23].

There are many reports on dietary patterns and mental health. The consumption of a ‘whole food’ (heavily loaded with vegetables, fruits, and fish) pattern [51] or grains–vegetables pattern [52] was linked with a reduced risk of depressive symptoms. A pro-inflammatory diet led to an increased risk of adverse mental health outcomes, including depressive symptoms, anxiety, and psychological well-being, according to a cross-sectional survey [20]. A prudent dietary pattern has been characterized by high intakes of vegetables, fruits, fish, legumes, poultry, and whole grains [53]. We expect that our findings are similar to these mentioned studies because the components of the RFS, based on the consumption of fruits, vegetables, grains, dairy products, and fish, are similar to a prudent dietary pattern.

The possible mechanisms underlying the impact of overall diet quality on mental health and QoL are explained via oxidative stress and inflammation. The diet strongly modulates genetic, immunological, biochemical, and neurodegenerative factors, and, consequently, is likely to impact on the development and occurrence of psychiatric illnesses [54]. An antioxidant-rich diet can act to slow or prevent aging-related pathophysiological and cognitive changes [55]. Inflammation can partially explain the associations with diet, medical illnesses, depression, and mortality [56–58]. The KNHANES observed that Korean elderly with a lower QoL are more likely to exhibit a lower consumption of vegetables and fruits and, thus, a lower intake of vitamins and minerals [59]. Another cross-sectional study emphasized the benefits of increasing nutrient intakes, including protein, vitamins, and minerals on HRQoL, in sarcopenic older adults [60].

The RFS is calculated based on the reported consumption of foods that contain antioxidant nutrients [29, 34], such as vitamins, selenium, and carotenoids, including β-carotene [61]. Lower diet quality may lead to inadequate antioxidant status and greater inflammation and, thus, negatively influence the pathophysiology of depressive illnesses [62] and the HRQoL [63]. Contrariwise, a better overall diet quality (higher HEI score), was shown to reduce inflammation, and improve health status, or functional outcomes affecting QoL in postmenopausal American women who survived early-stage breast cancer [64].

The current study had some limitations. First, due to the cross-sectional design, we could not confirm the causal relationship between the RFS and mental disorders or QoL. Second, only the RFS was used to assess the dietary quality, and no instruments were used for evaluating the total intake or intake of nutrients. Nevertheless, to the best of our knowledge, this study is the first to show a favorable association of the RFS with mental health and QoL in Korean adults. Spiritual well-being is strongly associated with all other domains of QoL (overall QoL, and mental, physical, emotional, and social well-being) [65]. Although the exact reason for the findings in this study is unclear, several explanations have been highlighted above. In any case, the current results infer the benefits of high diet quality on mental health and QoL, as investigated through standardized assessment tools for the diagnosis of depressive and anxiety disorders.

## Conclusions

This study demonstrates that better diet quality is associated with lower depressive symptoms and better QoL in Korean adult men and women. More rigorous prospective studies of large cohorts or intervention studies will be needed to investigate if diet quality can influence the maintenance of mental health and QoL over time in an adult population. These findings emphasize the importance of addressing overall diet quality in future community or population-based programs or in the development of policies to prevent mood disorders and support healthy aging.

## Data Availability

The datasets analyzed in the current study are available from the corresponding author on reasonable request.

## Abbreviations

BAI: beck anxiety inventory
BDI: beck depression inventory
BMI: body mass index
HEI: healthy eating index
HRQoL: health-related quality of life
KNHANES: Korea National Health and Nutrition Examination Survey
MDS: mediterranean diet score
RCT: randomized controlled trial
RFS: recommended food score
WHOQoL: World Health Organization quality of life
